# Identification of linear epitopes on the flagellar proteins of *Clostridioides difficile*

**DOI:** 10.1038/s41598-021-89488-7

**Published:** 2021-05-11

**Authors:** A. Razim, K. Pacyga, P. Naporowski, D. Martynowski, A. Szuba, A. Gamian, S. Górska

**Affiliations:** 1 Laboratory of Immunobiology of Microbiome, Hirszfeld Institute of Immunology and Experimental Therapy, PAS, Wroclaw, Poland; 2 Laboratory of Medical Microbiology, Hirszfeld Institute of Immunology and Experimental Therapy, PAS, Wroclaw, Poland; 3 Laboratory of Genomics and Bioinformatics, Hirszfeld Institute of Immunology and Experimental Therapy, PAS, Wroclaw, Poland; 4 Division of Angiology, Wroclaw Medical University, Wroclaw, Poland; 5 Department of Internal Medicine, 4th Military Hospital in Wroclaw, Wroclaw, Poland

**Keywords:** Infectious diseases, Vaccines, Vaccines

## Abstract

*Clostridioides difficile* (*C. difficile*) is an opportunistic anaerobic bacterium that causes severe diseases of the digestive tract of humans and animals. One of the possible methods of preventing *C. difficile* infection is to develop a vaccine. The most promising candidates for vaccine antigens are the proteins involved in the adhesion phenomena. Among them, the FliC and FliD are considered to be suitable candidates. In this paper, the FliC and FliD protein polypeptide epitopes were mapped in silico and by using PEPSCAN procedure. We identified four promising epitopes: ^117^QRMRTLS^123^, ^205^MSKAG^209^ of FliC and ^226^NKVAS^230^, ^306^TTKKPKD^312^ of FliD protein. We showed that ^117^QRMRTLS^123^ sequence is not only located in TLR5-binding and activating region, as previously shown, but forms an epitope recognized by *C. difficile*-infected patients’ antibodies. ^205^MSKAG^209^ is a *C. difficile*-unique, immunogenic sequence that forms an exposed epitope on the polymerized flagella structure which makes it a suitable vaccine antigen. ^226^NKVAS^230^ and ^306^TTKKPKD^312^ are well exposed and possess potential protective properties according to VaxiJen analysis. Our results open the possibility to use these epitopes as suitable anti-*C. difficile* vaccine antigens.

## Introduction

*C. difficile* is an opportunistic anaerobic bacterium that causes severe diseases of the digestive tract of humans and animals. It is a Gram-positive, rod-shaped pathogen producing dangerous to health toxins (TcdA and TcdB) and spores that can survive in high temperatures and generally used cleaning agents^1^. *C. difficile* is responsible for colitis and the occurrence of acute life-threatening diarrhea especially in hospitalized elderly and patients undergoing antibiotic treatment. Since the number of hypervirulent *C. difficile* strains as well as the strain resistance towards commonly used antibiotics is increasing, this bacteria is a serious problem that requires development of new prevention and treatment methods^2,3^.

One of the possible methods of preventing *C. difficile* infection (CDI) is to induce by vaccination specific antibodies directed against molecules involved in the adhesion process^4^. Blocking the binding sites on the surface of bacteria prevents the adhesion of pathogens to the epithelial cells of host and stops the development of infection. It has been shown that protein components of the surface of *C. difficile* have immunomodulatory properties^5–7^. Moreover, they contain conserved regions in their structure, thus they are ideal candidates for vaccine components/antigens. The most promising results of the immunization studies and serological analysis were obtained for proteins: Cwp66, Cwp84, FliC, FliD and Fbp68^5,8,9^. Simultaneously these antigens can be used to obtain protective and therapeutic antibodies.

Flagellum was shown to have an important role in adhesion of various pathogens and is one of the vital virulence factors^10–12^. Flagellin, the most abundant protein in the flagellum, is stimulating host immune system by interaction with Toll-Like-Receptor 5 (TLR5) and is considered to be a suitable vaccine adjuvant^13^. In *C. difficile*, the flagellum is composed of FliC (flagellin) and FliD (cap) proteins and both of them bind murine mucus^14^ and are implicated in the process of biofilm formation^15^. In contrast to non-flagellated or non-toxigenic strains of *C. difficile*, the flagellated strains induce inflammatory response synergistically with toxins in CDI mouse models^16^. It is well known that *C. difficile*-infected patients develop a robust antibody response to FliC and FliD proteins. What is particularly interesting is that the level of antibodies against flagella is lower in the *C. difficile*-infected patient group than in the control group which suggests their possible protective role^8^. It was previously shown that recombinant FliC is immunogenic and protective in a murine model of CDI and partially protective in CDI hamster model^17^. The study of restriction fragment length polymorphism of 17 clinical isolates of *C. difficile* showed high similarity of protein sequences of FliD and FliC between isolates^9^. Both flagella-building proteins of *C. difficile* are good candidates for use in vaccines in combination with suitable adjuvants due to their conservative nature and the ability of inducing a strong immune response.

To ensure vaccine safety each antigen has to be thoroughly characterized in terms of its cross-reactivity and possible autoimmunizing properties^7^. Using epitope-based vaccines is a well-recognized approach that allows to obtain safe and effective formulations. FliC and FliD have been proposed to be suitable as anti-*C. difficile* antigens. So far, there was no study designed to explore their epitopes using empirical methods. In this paper we describe the process of epitope mapping of two flagellar proteins from *C. difficile* using three types of patient sera. We identified the shortest amino acid sequences recognized by patient sera, tested their cross-reactivity with other clinically-relevant flagellated bacterial strains. We also bioinformatically defined the localization of identified epitopes in the assembled flagellum and evaluated the possibility of using flagellar epitopes as vaccine antigens.

## Methods

### Blood sera

#### Human blood sera

Serum samples from *C. difficile*-infected patients were provided by 4^th^ Military Clinical Hospital in Wrocław. Umbilical cord blood samples (*n* = 10) and peripheral blood samples from healthy volunteers (*n* = 16) were provided by internal collection of Hirszfeld Institute of Immunology and Experimental Therapy. All sera samples were collected and used under the written approval of Bioethics Committee of the Medical University of Wroclaw (no. KB-631/2015). An informed consent was obtained from all of the participants. Experiments were conducted in accordance with the Helsinki Declaration, 1975. In total, serum samples from sixteen patients suffering first episode of CDI were collected. Patients were diagnosed based on the following symptoms: three or more loose stools within 24 h, fever, abdominal pain, and positive test results for the presence of glutamate dehydrogenase, as well as CD toxins (C. Diff Quik Chek Complete; TECHLAB, Inc., Blacksburg, VA, USA)^18^. Within groups, the sera were pooled, aliquoted, and stored at − 20 °C until further used.

#### Animal blood sera

Serum samples of rabbits immunized with whole acetone-inactivated bacteria: *Escherichia coli* O56 PCM 2372 (*E. coli*), *Citrobacter freundii* PCM 1506 (*C. freundii*), *Hafnia alvei* PCM 1203 (*H. alvei*), *Shigella sonnei* (*S. sonnei*) were used in this manuscript. All of the above sera were already used in other experiments^19,20^ and were obtained from the sera collection of Hirszfeld Institute of Immunology and Experimental Therapy.

### Multiple sequence alignment

All used sequences were obtained from National Center for Biotechnology Information (NCBI, https://www.ncbi.nlm.nih.gov/) protein database^21^. Multiple sequence alignment and analysis of sequence homology were performed using Clustal Omega program (https://www.ebi.ac.uk/Tools/msa/clustalo/)^22^ and BLAST (https://blast.ncbi.nlm.nih.gov/Blast.cgi).

### Prediction of linear antigenic epitopes

The FliC (NCBI accession number: CBE01876) and FliD (NCBI accession number: CBE01872) sequences of *C. difficile* R20291 strain used for epitope prediction were obtained from NCBI protein sequence database. For prediction of pentadeca- and hexadecapeptide linear antigenic epitopes EPMLR tool and SVMTrip were used^23,24^.

### Peptide synthesis

NCP Block of 96 hydroxypropylmethacrylate pins and F-moc protected amino acids were obtained from Mimotopes (Clayton, Victoria, Australia). Chemicals used for synthesis and side chain deprotection like piperidine, dimethylformamide (DMF), trifluoracetic acid, diisopropylcarbodiimide (DIC) were of analytical grade, purchased from Merck Millipore (Burlington, MA, USA). Reagents like 2-mercaptoethanol, ethanedithiol, anisole, 1-hydroxybenzotriazole (HoAt), N,N-diisopropylethylamine (DIEA) and bromophenol blue were purchased from Sigma-Aldrich (Saint Louis, MO, USA).

A total of 20 FliC 15-amino acid- and 32 FliD 16-amino acid-long peptides covering predicted epitopes were synthesized. Peptides were synthesized according to standard protocol^25^ with slight changes^6,26^.

### Pin-bound modified ELISA

In order to test the interaction between serum antibodies and pin-bound synthetic peptides a modified ELISA was performed. 96-well polystyrene plates were used for all ELISAs. First, pins were incubated for 1 h in a solution of 1% bovine serum albumin (BSA, Sigma Aldrich) in TBS-T (Tris-buffered saline with 0.05% Tween20) at room temperature. Patients’ sera (or rabbit sera) in 1:1 000 dilution were used as primary antibodies source, incubated for 2 h at room temperature. In the next step pins were washed with TBS-T. Pins were incubated with secondary antibodies conjugated with alkaline phosphatase (anti-human 1:10,000 or anti-rabbit 1:30 000 IgG antibodies) for 1 h at room temperature (Sigma-Aldrich, cat. no A1543 and A3687). Pins were washed again and the color reaction was developed with AP Yellow for 30 min at room temperature. The reaction was stopped by removing pins from the solution and the colorimetric reaction was measured at 405 nm with a microplate reader (PowerWave HT, BioTek Instruments, Winooski, VT, USA). In the last step pins were regenerated by sonication for 10 min in disruption buffer preheated to 55 °C using ultrasonic bath (Cole Parmer, Bunker Court Vernon Hills, IL, USA). Pins were washed with MilliQ water preheated to 55 °C for 5 min. Pins intended for storing were additionally washed with methanol for 2 min and air-dry. All tests were performed at least in triplicate. The baseline for ELISA result analysis was counted as a mean of all readings obtained for *C. difficile*-infected patient group^26^.

### Recognition of minimal epitopes and essential amino acids

Peptide sequences which showed the highest antibody binding were mapped in detail to determine the shortest amino acid sequence needed for the activity. For each pentadeca- and hexadecapeptides a library of truncated sequences was synthesized. The library was prepared by synthesizing peptides with removed flanking residues of the original peptide.

In the next step, essential amino acids were recognized by alanine, glycine or valine scanning, the so-called alanine-walk^27^. Scanning was performed by synthesizing a set of peptides in which each of the following amino acids is replaced by an alanine, glycine or valine.

### Immunogenicity and autoimmunoreactivity

Epitopes identified by human sera were further evaluated using VaxiJen v.2.0 which is an alignment-independent tool predicting protein antigenicity basing on its physical properties^28^. Since two epitopes were to short for VaxiJen analysis, we added glycine at the C-end of the peptide for this analysis. Moreover, we searched the autoepitope database in order to confirm that the epitopes of FliC and FliD are not related to any known auto-epitopes^29^. We also looked for similar sequences in the known human protein amino acid sequences using BLAST.

### Epitope localization in flagella

A model of *C. difficile* FliC monomer was generated by Phyre2 server^30^ with 100% confidence. A FliD monomer was obtained with SWISS-MODEL^31^. The models of FliC and FliD were superimposed on a cryo-EM structure of *Pseudomonas aeruginosa* flagellar filaments (pdb 5wk6) and on a flagellar cap hexamer from *Escherichia coli* (pdb 5h5v) respectively. The interactions between TLR5 and *C. difficile* FliC were generated by sequence alignment of *C. difficile* model and *Salmonella enterica* protein (pdb 3v47) using Pymol^32^. All superpositions and protein figures were prepared with Pymol. We characterized the localization of epitopes in these proteins. Analysis of the protein domains was performed with Predict Protein software^33^.

### Statistical analysis

All graphs were prepared and data analyzed with GraphPad Prism 9. Unless otherwise stated data were analyzed using two-way ANOVA (p < 0.05). All measurements were performed at least in a triplicate. Pearson correlation coefficient (p < 0.05) was used to analyze the link between sequence similarity of *C. difficile* FliC and FliD epitopes to flagellar sequences of other bacterial strains and their immunoreactivity.

## Results

### Mapping of flagellar amino acid sequences with patients’ sera revealed highly immunoreactive regions

We designed a set of peptides for chemical synthesis that covered whole proteins amino acid sequences and overlaid in silico predicted epitopes (Supplementary Tab. S1). We synthesized 20 peptides based on FliC sequence from *C. difficile* R20291 strain that were 15-amino acid long (Supplementary Tab. S2). We synthesized 32 peptides based on FliD sequence from *C. difficile* R20291 strain that were 16-amino acid long (Supplementary Tab. S3). Peptides were analyzed in terms of their immunoreactivity with three types of sera: *C. difficile*-infected patients, healthy controls and umbilical cord blood sera. All of the used sera were diluted 1:1 000 as described in M&M section.

**Figure 1:**
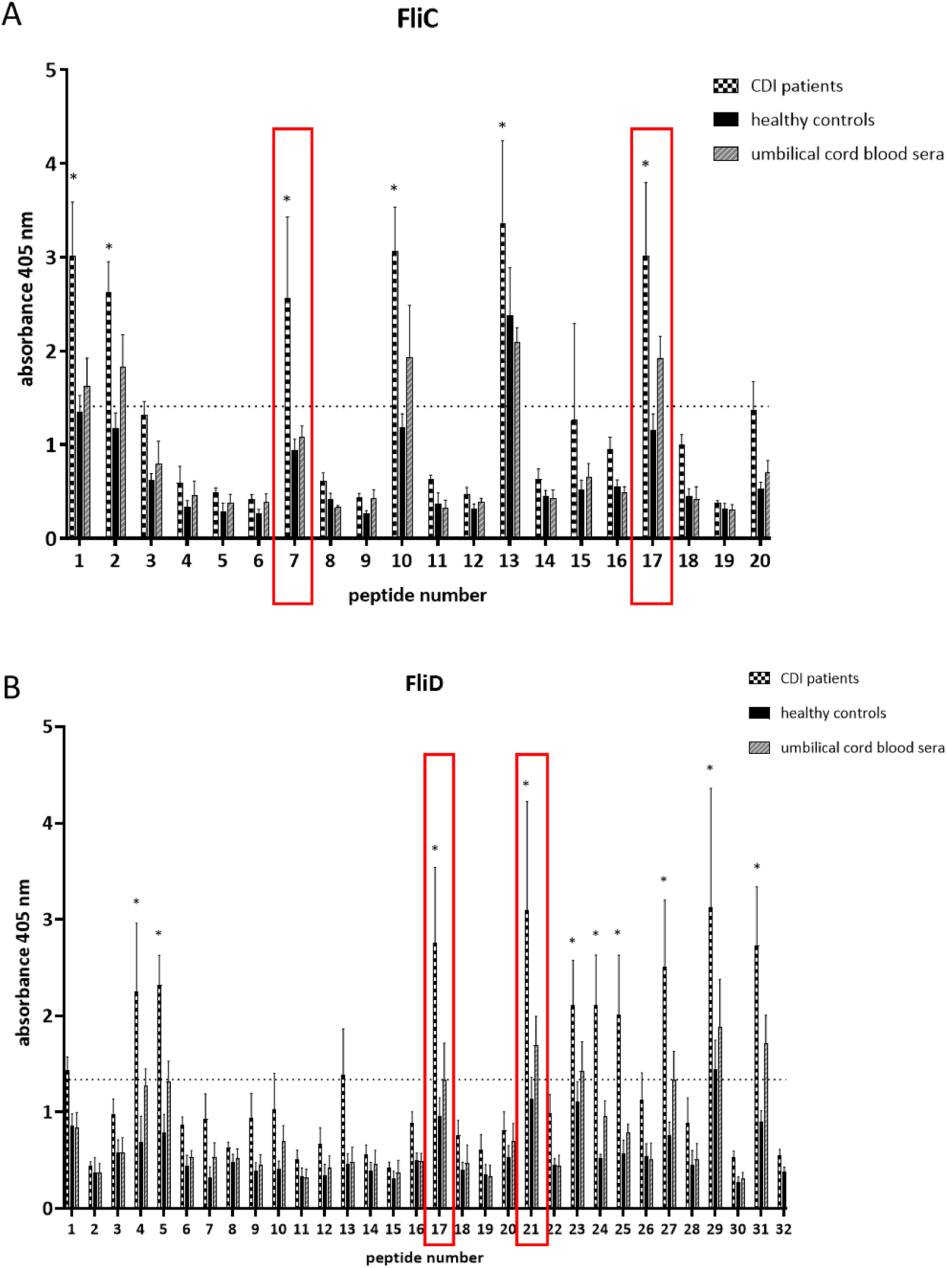
Immunoreactivity of peptides synthesized for mapping FliC (**A**) and FliD (**B**). Immunoreactivity was analyzed by pin-bound ELISA in which three groups of pooled sera: *C. difficile*-infected patients, healthy controls and umbilical cord blood sera were used in 1:1000 dilution. Anti-human IgG antibody conjugated to AP was used in 1:10,000 dilution. Assay was performed four times, the data shown is mean with ± SD. Statistical significance of the results obtained for *C. difficile*-infected patient group was calculated using two-way ANOVA in reference to the calculated baseline (1.41 for FliC and 1.34 for FliD) *p < 0.0332. Red brackets indicate peptides selected for further analysis.

**Figure 2:**
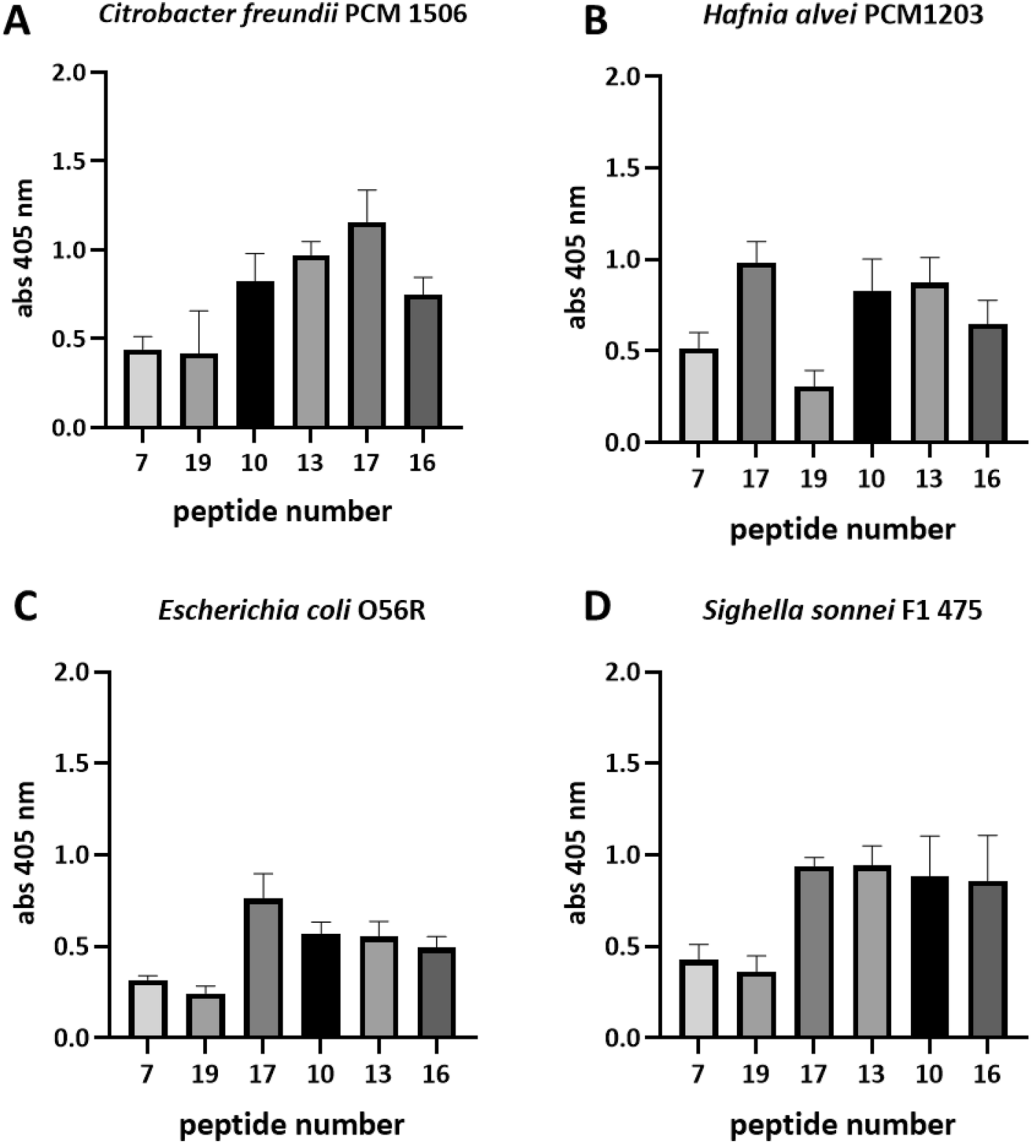
Cross-reactivity analysis of *C. difficile* FliC peptides and sera from rabbits immunized with other flagellated bacterial strains. *C. difficile* FliC peptides are organized from the ones with the lowest sequence homology to the flagellar sequences of bacteria used for rabbit immunizations to the ones with the highest sequence homology. Pin-bound ELISA was performed using rabbit sera (1: 1000) from animals immunized with whole acetone-inactivated bacteria. ELISA was performed at least three times. Data shown are means with ± SD.

Epitope mapping of pentadecapeptides synthesized based on FliC amino acid sequence resulted in two peptide candidates for detailed mapping which are NTSSIMSKAGITSST (FliC7) and NILQRMRTLSVQSSN (FliC17) (Fig. 1A). Overall, peptides were recognized at the highest level by IgG antibodies from sera obtained from patients during the initial symptomatic infection (*C. difficile*-infected patients). The same peptides were recognized at a much lower level by the sera of other groups (umbilical cord blood and healthy blood donors group). The baseline was calculated as the mean of all measurements obtained for *C. difficile*-infected patient group (mean absorbance = 1.41). Six of the tested peptides (FliC1, 2, 7, 10, 13, 17) were significantly more immunoreactive than the rest but not all of them were suitable for further analysis as vaccine epitopes because of their cross-reactivity with serum from rabbits immunized with other flagellated bacterial strains or high immunoreactivity with sera from the relapsed patient group (Fig. 2 and Supplementary Fig. S1) or because of their high immunoreactivity with sera from people without the history of CDI can be a result of some cross-reactivity. An interesting observation is that FliC11 which contains one of the bioinformatically predicted epitope in its sequence has a very low immunoreactivity. FliC7 and FliC17 were qualified by us for further detailed analysis.

Epitope mapping of hexadecapeptides synthesized based on FliD amino acid sequence resulted in two candidates for detailed mapping which were ASGNKVASVYGKNLEA (FliD17) and TTKKPKDYPPLTDAQK (FliD21) (Fig. 1B). As in the case of FliC, peptides were recognized at the highest level by *C. difficile*-infected patients’ sera group, then umbilical cord blood sera and at least by the healthy controls. The calculated baseline for *C. difficile*-infected patient group was 1.34. Ten of synthesized peptides were significantly more immunoreactive than the rest and were analyzed in terms of their cross-reactivity with serum from rabbits immunized with other flagellated bacterial strains. FliD1, 2 and 3 which were designed to cover bioinformatically predicted epitopes had a very low immunoreactivity with all three groups of tested sera. FliD17 and FliD21 were qualified by us for further detailed analysis because of their high immunoreactivity with CDI patient sera and low immunoreactivity with healthy sera.

### The level of amino acid sequence homology only partially determines peptide cross-reactivity

Simultaneously with epitope mapping of FliC7, FliC17, FliD17 and FliD21 peptides, we analyzed the cross-immunoreactivity of synthesized *C. difficile* FliC and FliD peptides with sera from rabbits immunized with other flagellated bacteria strains. We used sera from rabbits immunized with *C. freundii*, *H. alvei*, *E. coli* and *S. sonnei* in order to decipher the potential impact of peptide homology in their immune-reactivity. For both proteins we selected a set of peptides with varying sequence homology to FliC (Supplementary Fig. S2) and FliD (Supplementary Fig. S3) of the above bacterial strains and varying immunoreactivity. Cross-reactivity analysis performed by us shows that the level of amino acid sequence similarity is not always the best prognostic marker for possible cross-reactivity. For peptides based on *C. difficile* FliC we got a linear relationship between sequence similarity and cross-reactivity with *C. freundii* and *S. sonnei* vaccinated rabbits (Fig. 2a,d). However, when we counted the Pearson correlation coefficient for this data the visible relationship turned out to be statistically insignificant (Supplementary Tab. S4). Peptide FliC7 that had the lowest homology with flagellin of *C. freundii* (1/15 similar amino acids), *H. alvei* (1/15), *E. coli* (1/15) and *S. sonnei* (4/15) showed low cross-reactivity but most homological peptide FliC16 (9–10/15) was never the one with the highest level of cross-reactivity.

**Figure 3:**
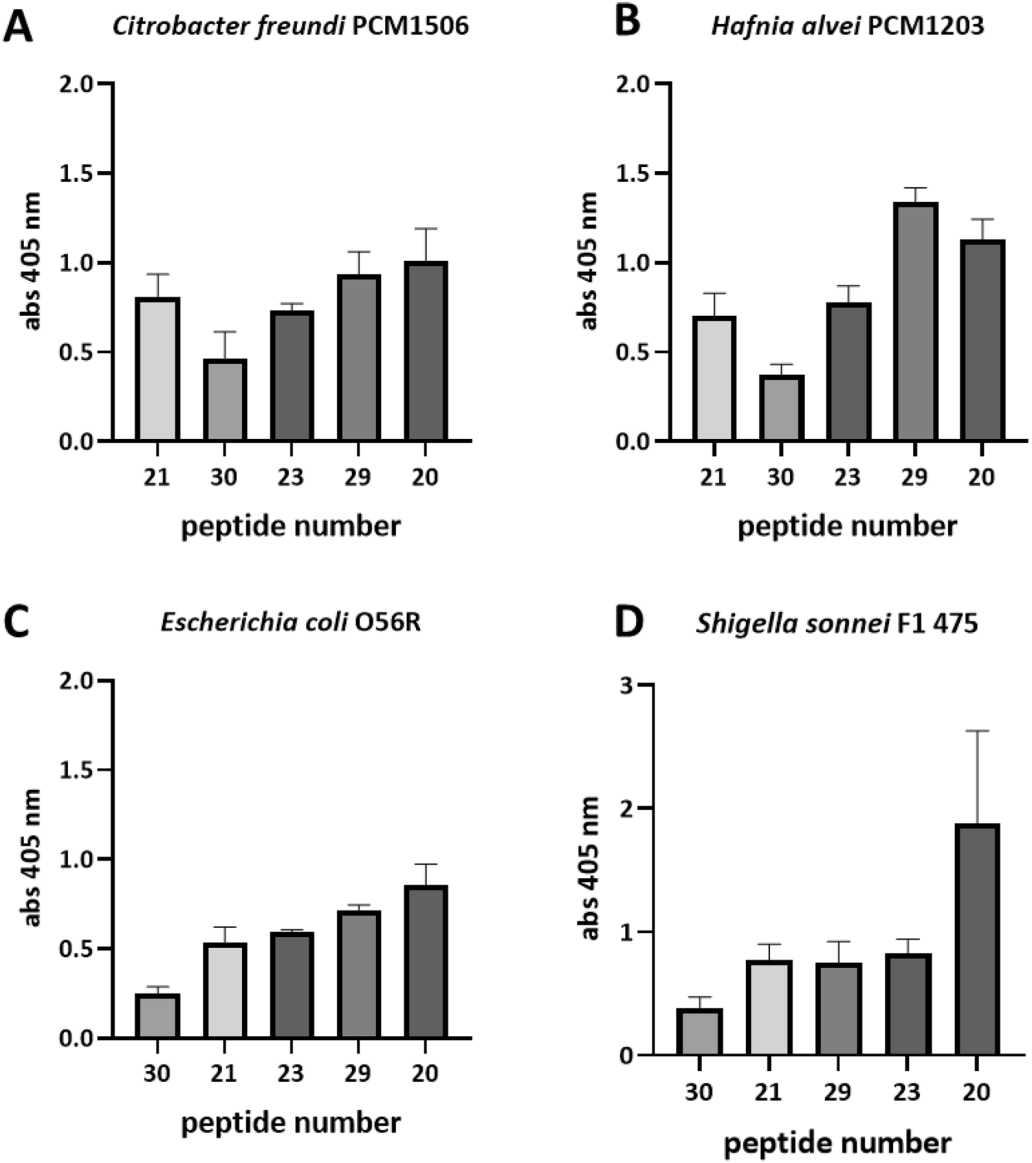
Cross-reactivity analysis of *C. difficile* FliD peptides with sera from rabbits immunized with other flagellated bacterial strains. *C. difficile* FliD peptides are organized from the ones with the lowest sequence homology to the flagellar sequences of bacteria used for rabbit immunizations to the ones with the highest sequence homology. Pin-bound ELISA was performed using rabbit sera (1: 1000) from animals immunized with whole acetone-inactivated bacteria. ELISA was performed at least three times. Data shown are means with ± SD.

In case of *C. difficile* FliD peptides there is a visible and statistically significant relationship between sequence similarity and cross-reactivity of two tested sera—*E. coli* (*p* = 0.0391) and *S. sonnei* (*p* = 0.0016) (Supplementary Tab. S4). It needs to be mentioned that the overall homology between *C. difficile* FliD protein and the corresponding proteins from other bacterial strains is very low as well as the cross-reactivity for most of the tested sequences. FliD16 has no homological sequence in *C. freundii* and *H. alvei*; FliD 30 has no homological sequence in *E. coli*. The highest homology was determined for FliD20 (6/16 of similar amino acids for *E. coli* and *H. alvei*; 7/16 for *S. sonnei*; 9/16 for *C. freundii*) (Fig. 3).

### Recognition of flagellar epitopes

**Figure 4:**
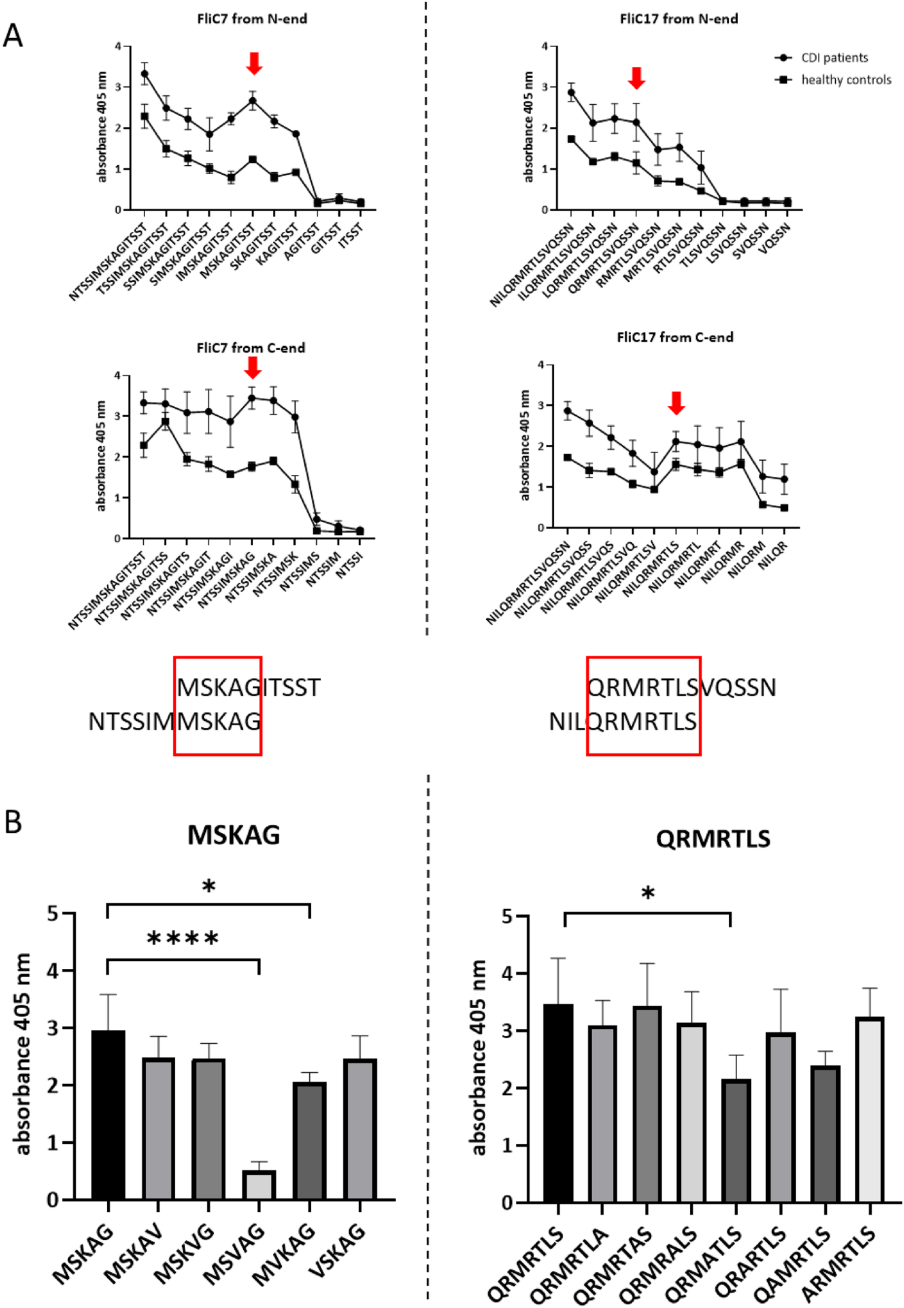
Mapping of *C. difficile* flagellin epitopes. (**A**) The analysis of the immunoreactivity of the library of truncated peptides of FliC7 and FliC17. Pin-bound ELISA was performed using pooled sera from *C. difficile*-infected patients and healthy volunteers (1:1000 dilution). Anti-human IgG conjugated with AP were used as secondary antibodies (1:10,000 dilution). Means with ± SD of three measurements are shown. (**B**) The “alanine walk” analysis of MSKAG and QRMRTLS epitopes. Pin-bound ELISA was performed using pooled *C. difficile*-infected patients’ sera (1:1000 dilution). Anti-human IgG conjugated with AP were used as secondary antibodies (1:10,000 dilution). Means with ± SD of three measurements are shown. Data analyzed with one-way ANOVA; *p < 0.0332; **p < 0.0021; ***p < 0.0002; ****p < 0.0001.

We mapped epitopes in two peptides of each of the flagellar proteins FliC7 and FliC17 from *C. difficile* flagellin and FliD17 and FliD21 from *C. difficile* cap protein. We synthesized a library of truncated peptides for each of the above sequences and analyzed their immunoreactivity with two types of patients’ sera (initial episode of CDI and healthy control sera). By this method, we were able to find the epitope in FliC7 which is ^205^MSKAG^209^ and in FliC17 which is ^117^QRMRTLS^123^ (Fig. 4A). In the next step we characterized the amino acids essential for antibody binding by “alanine walk” method. In epitope ^205^MSKAG^209^ each amino acid was substituted with valine since in the sequence there are both alanine and glycine, which are routinely used in this method. There is one essential amino acid in the ^205^MSKAG^209^ epitope which is lysine (Fig. 4B). Substitution of lysine in the ^205^MS**K**AG^209^ sequence results in total lost of epitope immunoreactivity. However, all of the other amino acids in the sequence are important for epitope reactivity since a substitution of each of them results in slight decrease in the level of immunoreactivity, especially in the case of serine. In epitope ^117^QRMRTLS^123^ there is no essential amino acid which substitution would lead to a total loss of epitope activity (Fig. 4B). However, there is a significant change in immunoreactivity when each of the two arginines in the sequences are substituted with alanine. Arginine as well as lysine belong to the group of positively charged amino acids.

**Figure 5:**
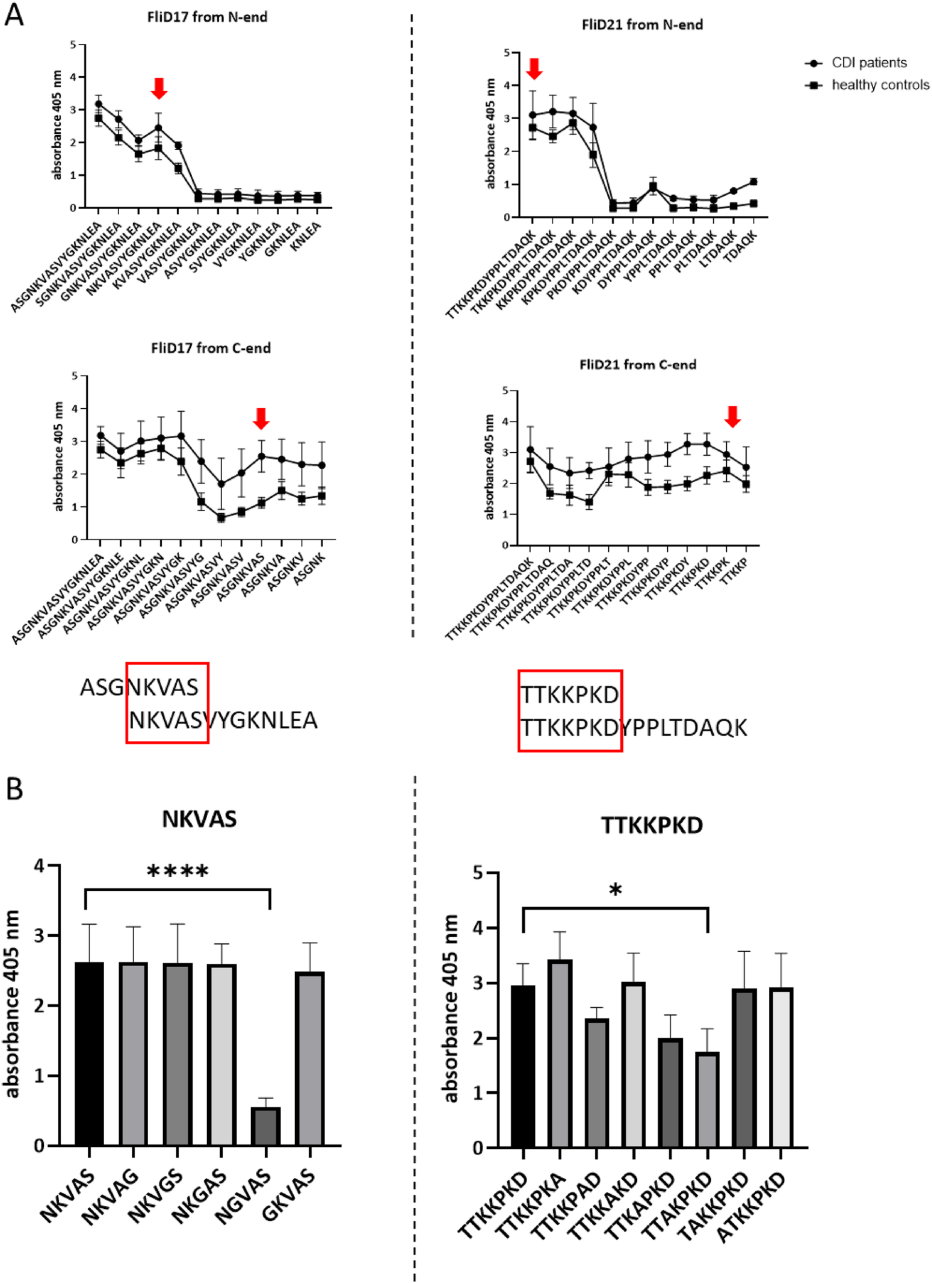
Mapping of *C. difficile* cap protein epitopes. (**A**) The analysis of the immunoreactivity of the library of truncated peptides of FliD17 and FliD21. Pin-bound ELISA was performed using pooled sera from *C. difficile*-infected patients and healthy volunteers (1:1000 dilution). Anti-human IgG conjugated with AP were used as secondary antibodies (1:10,000 dilution). Means with ± SD of three measurements are shown. (**B**) The “alanine walk” analysis of NKVAS and TTKKPKD epitopes. Pin-bound ELISA was performed using pooled *C. difficile*-infected patients’ sera (1:1000 dilution). Anti-human IgG conjugated with AP were used as secondary antibodies (1:10,000 dilution). Means with ± SD of three measurements are shown. Data analyzed with one-way ANOVA; *p < 0.0332, **p < 0.0021, ***p < 0.0002, ****p < 0.0001.

By using the library of truncated peptides we have found epitopes in FliD17 and FliD21 which are ^226^NKVAS^230^ and ^306^TTKKPKD^312^, respectively (Fig. 5A). Alanine walk method showed that in ^226^N**K**VAS^230^ epitope there is one essential amino acid which is lysine (Fig. 5B). The substitution of the rest of the amino acids did not change significantly epitope immunoreactivity. However, these amino acids might be important for epitope recognition by antibodies. In ^306^TT**KK**PKD^312^, again lysins are the most important for antibody binding. A substitution of each of them with alanine significantly changed epitope immunoreactivity.

### In silico analysis of epitopes immunogenicity and autoimmunoreactivity

In silico analysis of the potential immunogenic properties of FliC and FliD epitopes by VaxiJen v.2.0 (threshold > 0.4) showed that three of the epitopes have antigenic properties: ^205^MSKAG^209^(G) 0.6749, ^226^NKVAS^230^(G) 1.5113 and ^306^TTKKPKD^312^ 0.7361. The ^117^QRMRTLS^123^ epitope did not pass the test (-0.137). We performed a search in the database of known epitopes and autoepitopes, none of the epitopes characterized by us was found. Moreover, none of these sequences were found in known human protein sequences.

### Flagella modeling, epitope localization

**Figure 6:**
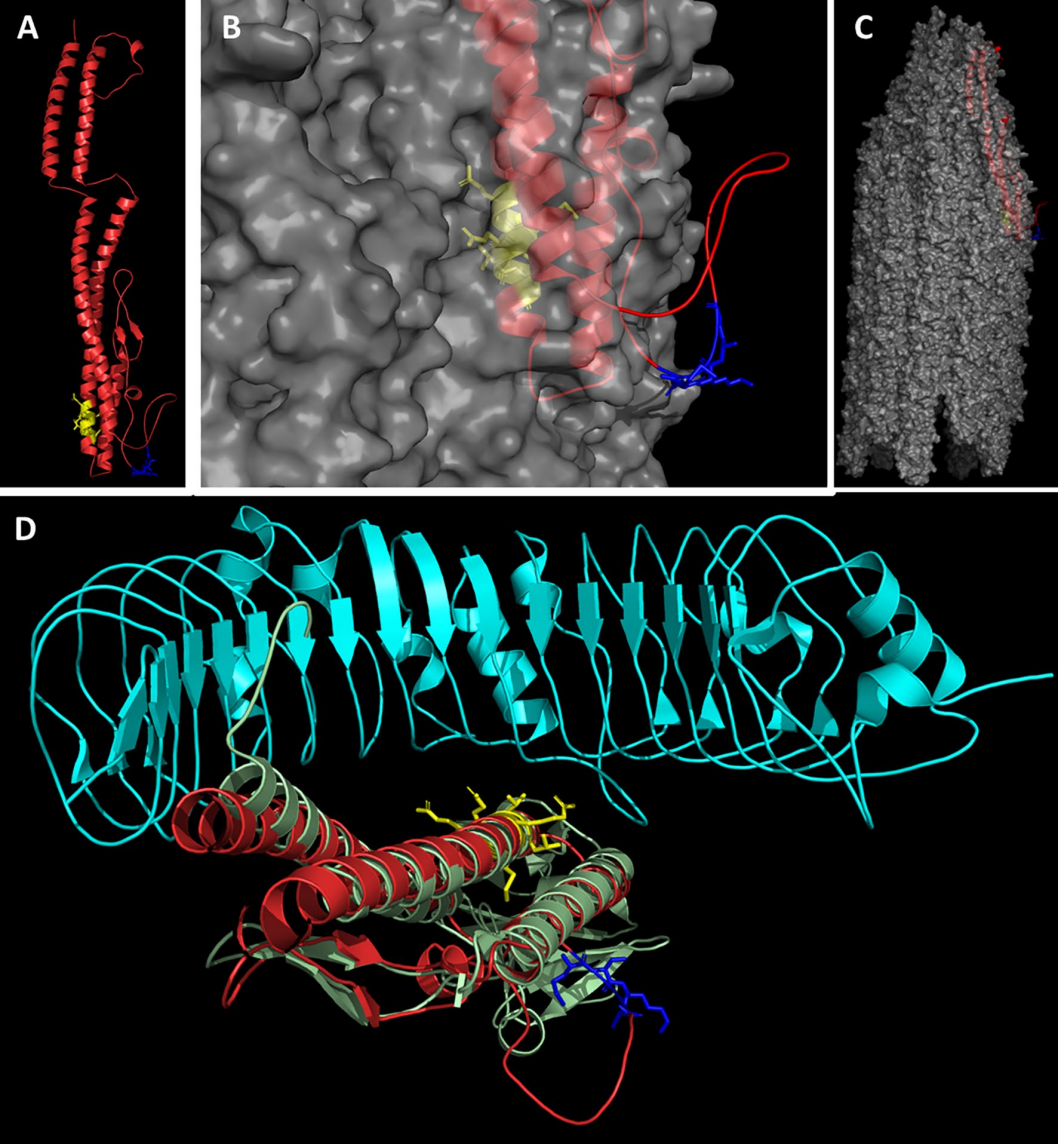
Modelling of *C. difficile* flagellin. (**A**) Secondary structure of FliC monomer. ^205^MSKAG^209^ epitope is indicated in blue. ^117^QRMRTLS^123^ epitope is indicated in yellow. (**B**, **C**) The FliC multimer which forms flagella body. (**D**) The interaction between TLR5 molecule (cyan) and FliC (green *Salmonella* FliC; red CD FliC). ^117^QRMRTLS^123^ epitope indicated in yellow in the center of the interaction.

**Figure 7:**
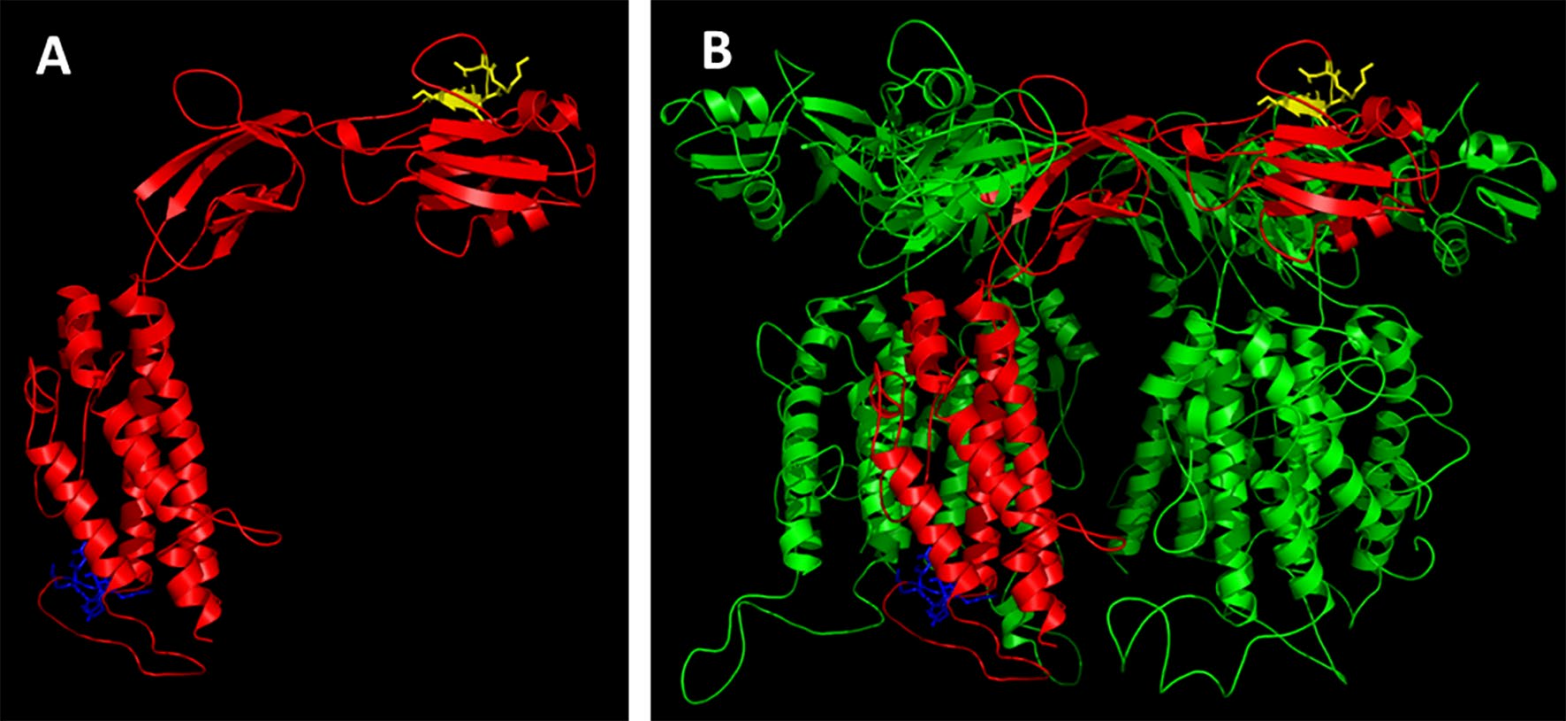
Modelling of *C. difficile* flagellar cap protein. (**A**) Secondary structure of FliD monomer. ^226^NKVAS^230^ epitope is indicated in yellow. ^306^TTKKPKD^312^ epitope is indicated in blue. (**B**) The FliD multimer that forms *C. difficile* flagellar cap.

We modeled the structure of FliC and FliD as monomers to localize the epitopes deteremined by us (Figs. 6A and 7A). Also, we modeled the structure of assembled flagella and flagellar cap hexamer to localize epitopes in the native bacterial protein structures (Figs. 6B,C and 7B). In the FliC protein one of the epitopes which is ^205^MSKAG^209^ is exposed to the environment and forms a loop which is usually easily accessible by antibodies. The ^117^QRMRTLS^123^ is buried inside flagella channel. We modelled the interaction between TLR5 and FliC based on crystallization data obtained for TLR5 and FliC from *Salmonella enterica* subsp. enterica serovar Typhimurium str. LT2. The results show that ^117^QRMRTLS^123^ is placed directly in the area of interaction between these two molecules.

In FliD, both epitopes are localized in structures other than alpha-helix which makes them more accessible for antibodies. FliD protein subunits form a hexamer and the two epitopes are localized on the two opposite sides of the hexamer. The analysis of FliD domains by Predict Protein server showed that ^306^TTKKPKD^312^ epitope is localized in the protein-binding domain.

## Discussion

Flagella is a highly conserved virulence factor for many pathogens including *Pseudomonas aeruginosa*^34^, *Salmonella* strains^35^ or *C. difficle*^36^. Flagella is not only indispensable for pathogen motility but also takes part in attachment to the host cells by acting as an adhesion molecule; participates in biofilm formation; and interacts with hosts’ immune cells, for example via Toll-like receptor 5 recognition^37^. Anti-flagella humoral response was shown to increase antibacterial function of host immune cells by phagocytosis stimulation^35^. Because of the above, flagella was already proposed as a suitable vaccine target^38^ and some of these vaccines already reached clinical trials^39^. Flagellar proteins were also proposed as potential adjuvants since they elicit potent, mixed Th1/Th2 immune response^40,41^. Moreover, Bruxelle et al. proposed *C. difficile* FliC as a mucosal adjuvant in an anti-*C. difficile* vaccine^42^. The aim of our work was to identify the epitopes of both FliC and FliD proteins, which could be suitable for specific subunit vaccine development.

The humoral response of the host against *C. difficile* was extensively studied^8,9^. In the context of *C. difficile* flagella, it has been shown that the antibody levels against FliC and FliD were higher in the control group compared to the *C. difficile* positive group, which indicate their potentially protective role^8,9^. Compared to results of Péchiné et al.^8,9^ we do not see such a relationship in our data, probably due to the fact that we have obtained sera at the earlier stage of infection and we measured the reactivity of specific anti-peptide IgG only instead of total anti-*C. difficile* immunoglobulins in patients’ sera (Fig. 1). It is also worth mentioning that high level of antibodies reacting with *C. difficile* proteins might be a result of cross-reactivity. We observed a higher immunoreactivity of umbilical cord blood sera than healthy controls sera which was also noticed in our previous studies^6,7,26^. It is well known that cord blood serum has a significantly different composition in terms of antibodies than blood circulating in the system and can be a source of potentially protective antibodies^43^. Based on the high immunoreactivity of *C. difficile*-infected patients’ sera and umbilical cord blood sera we were able to select several peptide sequences of FliC and FliD for further detailed analysis. Using bioinformatic and empirical methods, we were able to define the shortest peptide sequences involved in antibody binding and map the functional epitopes which consist of amino acids essential for epitope recognition by paratope (Figs. 4 and 5). We identified two epitopes in FliC protein, the main protein constituting the flagellar body. *C. difficile* FliC, like in many other Gram-positive bacteria, is composed of D0 and D1 domains required for TLR5 activation and flagellar body assembly^44^. ^117^QRMRTLS^123^ epitope identified by us using *C. difficile*-infected patients’ blood sera (Fig. 4) is localized in a conserved hot spot responsible for binding and activation of TLR5, complementary to the LRR9 loop of TLR5 identified by Song et al.^45^. The first arginine in the ^117^QRMRTLS^123^ sequence (R118) is highly conserved in TLR5-activating flagellins and its substitution with alanine have significantly reduced TLR5 activation by ten fold^45^. Song et al. suggests that this specific arginine mutation changes shape and chemical complementarity between flagellin and TLR5 LRR9 loop. Likewise Song et al.^45^, we observed the same effect in immunoreactivity of ^117^QRMRTLS^123^ after R118 substitution with alanine (Fig. 4B). However, Song et al. observed also a lower TLR5-binding effect in case of the mutation of L122 (two fold), similar effect was not shown by us in case of ^117^QRMRTLS^123^ immunoreactivity. Flagella activates TLR5 only in a monomeric form since sequences that are responsible for binding are buried in the assembled flagella^46^. The ^117^QRMRTLS^123^ epitope is localized in the TLR5-binding region buried inside flagella body which was also shown by us in this study (Fig. 6B,D). Despite the fact that ^117^QRMRTLS^123^ epitope has interesting properties, i.e. it is located in the TLR5 binding region and is recognized by patients' antibodies, its use in a vaccine should be approached with caution as it shows a high level of cross-reactions (Fig. 2, ^117^QRMRTLS^123^ is a part of peptide 17, see also Supplementary Tab. S2).

The second of epitopes identified by us in FliC ^205^MSKAG^209^ is well exposed in the assembled flagella and easily accessible by antibodies. Moreover, it is a part of VALVNTSSIMSK sequence which was shown to be glycosylated^47^. Glycosylation may have a broad effect on epitopes ranging from its inactivation to being necessary for its recognition by antibodies^48^. However, even without the glycosylation, ^205^MSKAG^209^ epitope is very well recognized by *C. difficile*-infected patients’ antibodies. The importance of ^205^MSKAG^209^ sequence for flagella functioning is unknown. Mapping of the functional epitope of ^205^MSKAG^209^ revealed that substitution of K207 with valine resulted in total loss of epitope immunoreactivity. It has been already shown by us that positively-charged lysine is critical for epitope-paratope interaction^26^. Since this epitope is characteristic for *C. difficile* and was positively verified by VaxiJen v2.0 as potentially immunogenic we plan to check its protective properties using mice model of CDI.

*C. difficile* FliD is a highly conserved protein that functions as a flagellar cap^49^. Antibodies specific for FliD are present in 87% of patients with diagnosed CDI which is a higher number than the one tested for FliC (21%) or *C. difficile* toxins A (60%) and B (25%)^8^. This means that FliD is highly immunogenic and might have a tremendous role in the effective humoral response against *C. difficile*. We mapped epitopes of *C. difficile* FliD using *C. difficile*-infected patients’ sera. The 16-amino acid peptides designed for FliD mapping showed similar level of immunoreactivity as the ones synthesized for FliC (Fig. 1A vs B). Also, FliD-derived peptides were more immunoreactive with umbilical cord blood sera than healthy volunteers sera which indicates that the antibodies contained in the serum of umbilical cord blood are more concentrated, at least at the time of birth when the blood was sampled by us. This is in line with findings of de Voer et al. who tested 197 maternal and cord blood pairs and showed that umbilical cord blood antibody titers against protein antigens from selected pathogens were 1.6 times higher than those in maternal blood^50^. With our approach we were able to find epitopes of *C. difficile* FliD which are ^226^NKVAS^230^ and ^306^TTKKPKD^312^. In each of them lysins were the most important amino acids for antibody binding (Fig. 5). Both passed the VaxiJen v2.0 test as immunogens and are well exposed in the FliD monomer and hexamer (Fig. 7). The analysis of FliD sequence with PredictProtein tool showed that ^306^TTKKPKD^312^ is localized in a protein-binding region. The *C.* difficile FliD hexamer forms table-like structure probably similar to the one crystalized for *Pseudomonas aeruginosa*^51^. According to structures shown by Postel et al. and our model of *C. difficile* FliD ^226^NKVAS^230^ is located in the ‘head’ region of FliD and ^306^TTKKPKD^312^ in the ‘leg’ region of this protein which is highly flexible and probably involved in protein oligomerization. The importance of these regions localization and their functions in the context of functional flagella assembly will be further investigated.

Another aspect of our work is the potential cross-reactivity of FliC and FliD peptides with host proteins or molecules present in its microbiome. Current solutions are based on the comparison of amino acid sequences. However, given that the interaction between epitope and paratope takes place at the atomic level and not amino acid residue level^52^, a bioinformatic-based approach may not always detect potential unwanted cross-reactions. Cross-reactivity analysis conducted in this study between *C. difficile* FliC and FliD peptides and sera obtained from rabbits immunized with flagellated bacteria strains showed that even a very small sequence homology can give potentially unwanted cross-reactions, and in turn, peptides with high homology to the antigen used for immunizations do not have to be highly immunoreactive (Figs. 2 and 3). These disparity may be due to the fact that the same antigens can immunize various organisms differently (human *vs* rabbit)^53^. Therefore, given that most studies are conducted on mice and then on humans, these differences should be taken into account. However, in some cases cross-reactivity might have good effects. Studies show that vaccination with pneumococcal vaccines might protect against SARS-CoV-2 infection and death^54^. Pvs48/45 antigens from *Plasmodium vivax* and *P. falciparum* share 55% identity and showed high cross-reactivity and cross-boosting properties^55^. This research gives hope for new vaccine targeting transmission of both species of malaria parasites which are responsible for the majority of malaria infections in the world. However, there are numerous reports linking molecular mimicry, resulting cross-reactions and autoimmunity with vaccination^56^. Solving this problem is particularly important in the era of increasing vaccine hesitancy to which the vaccine safety concern is one of the strongest argument. We propose that not only pathogen-unique peptides/structures should be considered to use as a vaccine antigen but also they should be thoroughly tested empirically in terms of possible cross- and auto-immunoreactivity. This is particularly important in the context of the use of the entire flagella protein as an adjuvant.

Within this paper, using bioinformatics methodology together with the analysis of patient’s sera immunoreactivity, we identified four epitopes of *C. difficile* flagella proteins. Our approach is in line with the currently recommended algorithm of searching for new candidates for vaccine antigens that advises the use of amino acid sequences with very low homology to self-antigens^57^, and we even extend our investigations with the analysis of cross-reactions that may occur between seemingly completely different sequences. We showed that ^117^QRMRTLS^123^ sequence identified by us in *C. difficile* flagellin is not only TLR5-binding and activating, as previously shown, but forms an epitope recognized by *C. difficile*-infected patients’ antibodies. ^205^MSKAG^209^ is a *C. difficile*-unique, immunogenic sequence that forms an exposed epitope on the polymerized flagella structure which makes it suitable vaccine antigen. ^226^NKVAS^230^ and ^306^TTKKPKD^312^ are two FliD epitopes, which are well exposed and protective according to VaxiJen analysis. Further, their potential protective properties will be verified by us in a mouse model of CDI.

## Supplementary Information

Supplementary Information

## Data Availability

The datasets generated during and/or analyzed during the current study are available from the corresponding author on reasonable request.
