# Supplementary material for: Identification of linear epitopes on the flagellar proteins of Clostridioides difficile
Source: Sci Rep. 2021 May 11;11:9940. doi: 10.1038/s41598-021-89488-7 (PMC8113543; doi:10.1038/s41598-021-89488-7)
Supplement: Supplementary file 1 — Supplementary Information [file 41598_2021_89488_MOESM1_ESM.pdf]

SUPPLEMENTARY DATA

Identification of linear epitopes on the flagellar proteins of *Clostridioides difficile*

Razim A.[1*], Pacyga K.[1], Naporowski P.[2], Martynowski D.[3], Szuba A.[4,5], Gamian A.[2], Górska S.[1]

[1] Laboratory of Immunobiology of Microbiome, Hirszfeld Institute of Immunology and Experimental Therapy, PAS, Wroclaw, Poland

[2] Laboratory of Medical Microbiology, Hirszfeld Institute of Immunology and Experimental Therapy, PAS, Wroclaw, Poland

[3] Laboratory of Genomics & Bioinformatics, Hirszfeld Institute of Immunology and Experimental Therapy, PAS, Wroclaw, Poland

[4] Division of Angiology, Wroclaw Medical University, Wroclaw, Poland

[5] Department of Internal Medicine, 4th Military Hospital in Wroclaw, Wroclaw, Poland

Table S1 Epitope sequences predicted in FliC and FliD by bioinformatic tools (SVMTriP and EPMLR).

FliC	
Score	Epitope
0.94597	AAESRIRDTDVASEM
0.9163	TKEGKIMRVNTNVSA
FliD	
Score	Epitope
1.000	RVTGLSGNFDMEGIIE
0.992	DEHGRVTHISKEQNSF
0.967	TIVSKINSLCADNDIK

Table S2 Peptide sequences synthesized for FliC. Predicetd epitopes indicated with red color.

PEPTIDE NO	PEPTIDE SEQUENCE
1	TKEGKIMRVNTNVSA
2	LIANNQMGRNVNAQS
3	ADELLQLKDEVERIS
4	SSIEFNGKKLLDGSS
5	TEIRLQVGANFGTNV
6	AGTTNNNNEIKVALV
7	NTSSIMSKAGITSST
8	IASLNADGTSGTDAA
9	KQMVSSLDVALKELN
10	TSRAKLGAQQNRLES
11	AAESRIRDTDVASEM
12	MLAQANQQPQGVLQL
13	KSMEKLSSGVRIKRA
14	ADDAAGLAISEKMRA
15	QIKGLDQAGRNVQDG
16	ISVVQTAEGALEETG
17	NILQRMRTLSVQSSN
18	ETNTAEERQKIADEL
19	STQNNLNNTIENVTA
20	VNLSKMNILVQASQS

Table S3 Peptide sequences synthesized for FliD. Predicted epitopes indicated with red color.

PEPTIDE NO	PEPTIDE SEQUENCE
**1**	RVTGLSGNFDMEGIIE
**2**	TIVSKINSLCADNDIK
**3**	DEHGRVTHISKEQNSF
**4**	ASMIRDKEKVDKAKQE
**5**	QQIVKWKQEIYRNVIQ
**6**	ESKDLYDKYLSVNSPN
**7**	SIVSEKAYSSTRITSS
**8**	DESIIVAKGSAGAEKI
**9**	NYQFAVSQMAEPAKFT
**10**	IKLNSSEPIVRQFPPN
**11**	ASGASSLTIGDVNIPI
**12**	IPISEQDTTSTIVSKI
**13**	SEQDTTSTIVSKINSL
**14**	ASYSEMTGELIISRKQ
**15**	TGSSSDINLKVIGNDN
**16**	LAQQIANDNGITFAND
**17**	ASGNKVASVYGKNLEA
**18**	NIDNIDYNVNSKGTAK
**19**	LTSVTDTEEAVKNMQA
**20**	FVDDYNKLMDKVYGLV
**21**	TTKKPKDYPPLTDAQK
**22**	EDMTTEEIEKWEKKAK
**23**	EGILRNDDELRGFVED
**24**	IQSAFFGDGKNIIALR
**25**	KLGINESENYNKKGQI
**26**	SFNADTFSKALIDDSD
**27**	KVYKTLAGYSSNYDDK
**28**	GMFEKLKDIVYEYSGS
**29**	STSKLPKKAGIEKTAS
**30**	ASENVYSKQIAEQERN
**31**	ISRLVEKMNDKEKRLY
**32**	AKYSALESLLNQYSSQ

Table S4 Analysis of correlation between peptides sequence homology and peptides immunoreactivity. Data analyzed by Pearson method. Table shows calculated p-values (significant p<0.05). Significant correlation indicated with an asterisk.

BACTERIA STRAIN	FLIC PEPTIDES	FLID PEPTIDES
Citrobacter freundii	0.1282	0.1866
Hafnia alvei	0.7525	0.4813
Escherichia coli	0.3884	0.0391*
Shigella sonnei	0.1015	0.0016*

Table S5 Sequence homology of flagellated bacteria strains and *Clostridioides difficile*. Sequences compared using ClustalOmega software.

BACTERIA STRAIN	FLIC	FLID
Citrobacter freundii	42.27%	18.32%
Hafnia alvei	42.65%	18.89%
Escherichia coli	42.96%	23.53%
Shigella sonnei	44.17%	21.57%

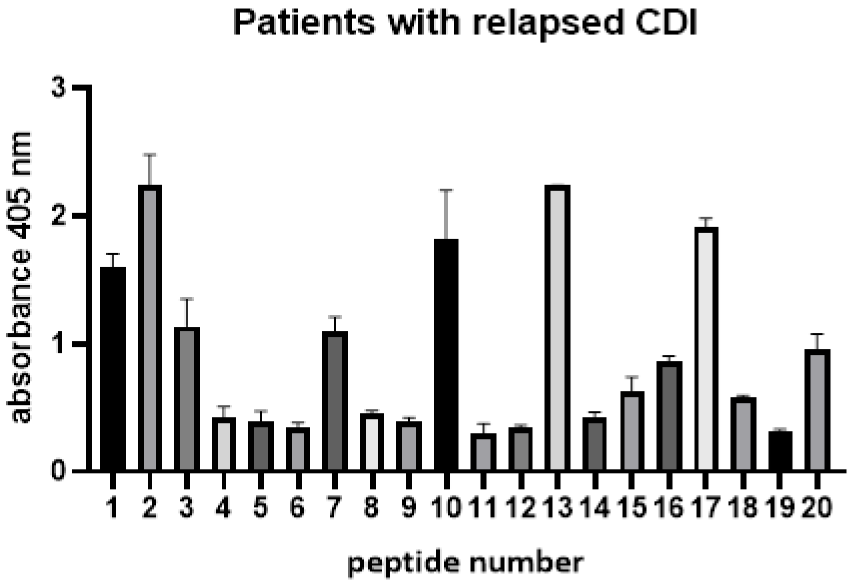

Figure S1 Immunoreactivity of FliC peptides with the pooled sera of relapsed patient group (n = 10). Performed using pin-bound ELISA, repeated four times; means with SD.

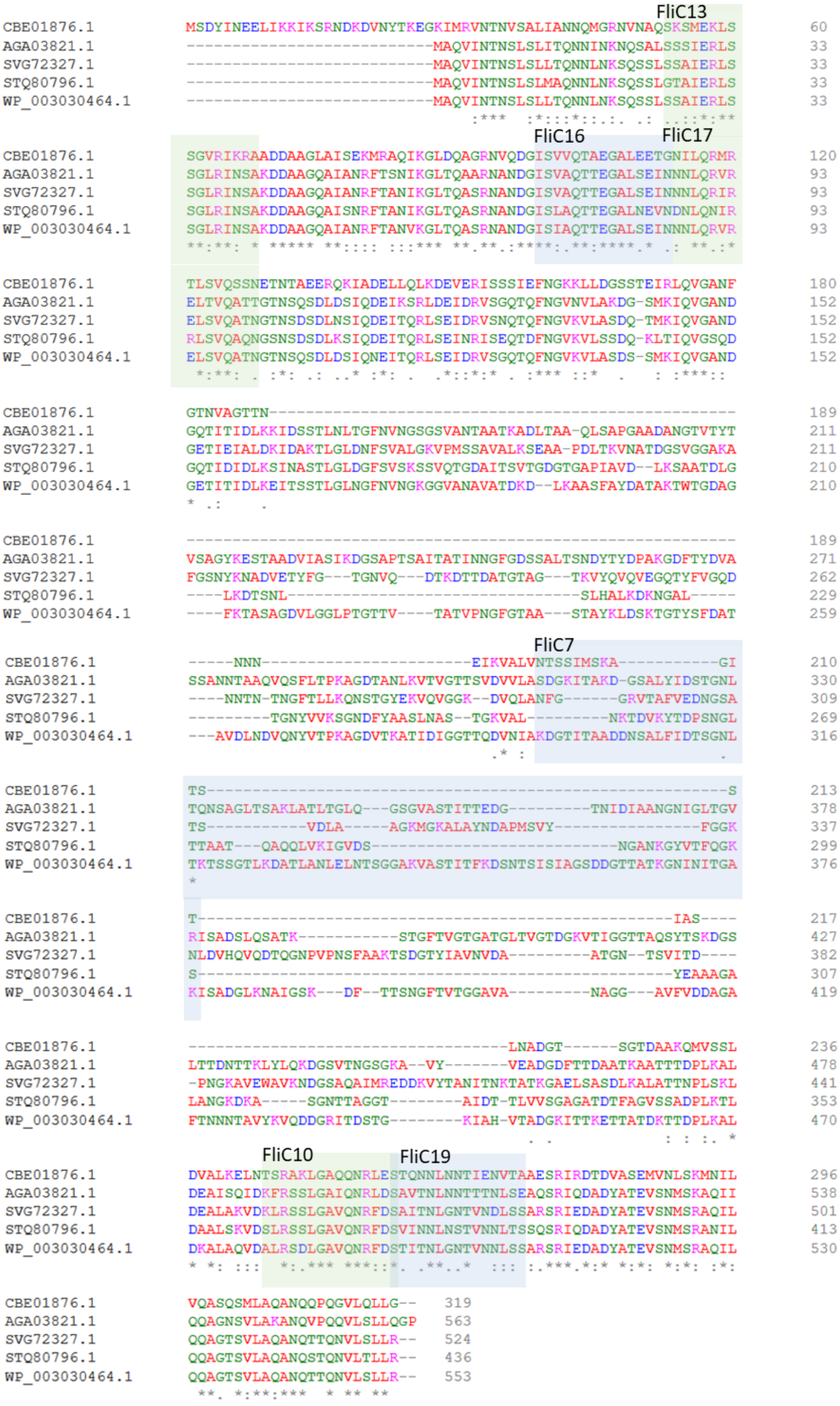

Figure S2 Analysis of sequence homology of FliC proteins from different flagellated bacteria strains. NCBI strain annotations: CBE01876.1 *C. difficile*; AGA03821.1 *E.coli*; SVG72327.1 *S. sonnei*; STQ80796.1 *H. alvei*; WP_003030464.1 *C. freundii*.

```
CBE01872.1          MSSISPIRVTGLSGNFDMEGIIEASMIRDK-EKVDKAKQEQQIVKWKQEIYRNVIQESKD    59
WP_000146770.1      MASISSL---GVGSGLDLSSILDSLTAAQ-KATLTPISNQQSSFTAKLSAYGTLKSA---    53
WP_094313017.1      MASFTSL---GVGSNLPLDTLLNNLTIAE-KKRLNPITQQQSDNTARLTAYGTLKSA---    53
WP_057101173.1      -----MI---NPR------TMAKAAAYADIATQSANLQQQQAELNAESSGLDSLSTA---    43
WP_046449645.1      MSDFTSI---DPQ------TMATQLASYDVMALQAALKKQQTSLTGQQDALKALKSA---    48
                          :   .          :       :       ::*   . .     :

CBE01872.1          LYDKYLSVNSPNSIVSEKAYSSTRITSSDESIIVAKGSAGAEKINYQFAVSQMAEPAKFT    119
WP_000146770.1      -LTTFQTANTALSKADL--FSATSTTSSTTAF-SATTAGNAIAGKYTISVTHLAQAQTLT    109
WP_094313017.1      -LVKFQTANTALNKADL--FKSTTVTSSNEDL-KVSTEAGAAPGIYTISVTQLAQAQSLR    109
WP_057101173.1      -LTDFQSAIDALNSDTDGPLTFSATS-NNDSA-TVSANSEAQAGTYSFYVSKLAQGQQST    100
WP_046449645.1      -MTDFRTALTALNKTNNGLLTNKVTTSLDNIA-NVTANSNATKGTYNLYVEQLAGSHQVA    106
                      : :.     .        . .  :          ..   . *     * : * ::*

CBE01872.1          IKLNSSEPIV-RQFPPNASGASSLTIGD------VNIPISEQDT-------TSTIVSKIN    165
WP_000146770.1      TRTTRDD--TKT---AIATSDSKLTIQQGGDKDPISIDISAANSSL------SGIRDAIN    158
WP_094313017.1      TDSPTIIASTKDALGDESSDTRTIKITQDGRKEPLEIKLNKDQTSL------DEISKTIN    163
WP_057101173.1      FQMT--DNFY------EATGTFDLTMED---GSSMEIDLSTADSDGDGYIDATGLVDAI-    148
WP_046449645.1      FDDMTDDAVN------NATGTFTLEVN----GKSIDIEM-------DGLETMSDLARAIN    149
                                 ::.   : :         :.* :              :   *

CBE01872.1          SL----CADNDIKASYSE-MTG--ELIISRKQTGSSSDINLKVIGNDNLAQQIANDNGIT    218
WP_000146770.1      ------NAKAGVSASIINVGNGEYRLSVTSNDTGLDNAMTLSVSGDDALQSFMGYDASA-    211
WP_094313017.1      ------DADSGISASIVKVKDGDYQLVLTA-SEGLANKMTISVEGDSKLNDLLAYDSKT-    215
WP_057101173.1      ---NSSDDNPGVSAALVK-TDGQTTIMLTSDTTGAQSGFTAT--VNGTV-----------    191
WP_046449645.1      KTNDGSEKSPAITASLIR-TDGEVKLMLSSDKTGAENELKLSGDIPAEM-----------    197
                              .  :.*:  .    *    : ::    *   . :. .        :

CBE01872.1          FANDASGNKVASVYGKNLEADVTDEHGRVTHISKEQNSF----NIDNIDYNVNSK-----    269
WP_000146770.1      ----SSNGMEVSVAAQNAQ---LT----VNNVAIENSSNTISDALENITLNLNDVTT---    257
WP_094313017.1      ----NTGNMKELVNAQNAQ---LN----VNGIDIERSSNKITDAPQGVTLDLTKKVT---    261
WP_057101173.1      ----IASPETAMTTPQDAV---INLGGKD-GPAITSSSNTFDDVIPGVTMTFTEVSDPDD    243
WP_046449645.1      ----DSGKKTTISEAKDAI---VYLGGNDTGLKITNSTNKLDGVIDGVTVELNQAQKVGD    250
                         :.         ::                    .:       .:   ...

                                                FliD20                FliD21
CBE01872.1          --GTAKLTSVTDTEEAVKNMQAFVDDYNKLMDKVYGLVTTKKPKDYPPLTDAQKEDMTTE    327
WP_000146770.1      --GNQTLTITQDTSKAQTAIKDWVNAYNSLIDTFSSLTKYTAVD---AGADS--------    304
WP_094313017.1      --D-VRVTVTKSNDKATEAIKGWVDSYNSLIDTFNTLTKYKEVD---PGAEA--------    307
WP_057101173.1      TSDVTTITVAEDSSASQAKVQTFVDAYNTLVDTVDSLTSNG----------G--------    285
WP_046449645.1      A--PLRINVNTDTSETETQVKAFIDAYNTMRDSLGKLTASG----------S--------    290
                        :.    ... :    :: ::: **.: *..   *.              .

                              FliD23
CBE01872.1          EIEKWEKKAKEGILRNDDELRGFVEDIQSAF--FGDGKNIIALRKLGINESENYNKKGQI    385
WP_000146770.1      ------QNSSNGALLGDSTLRTIQTQLKSMLSNTVSSSNYKTLAQIGITTD---PSDGKL    355
WP_094313017.1      ------QDKNNGALLGDSVVRTIQSGIRAQFANGASDGAFKTLNEIGIKQD---GTTGKL    358
WP_057101173.1      ------DGSNPGVFAGDAGISSLTNQLDDIAHSYYDG---VSIVDYGISL----DSDGHL    332
WP_046449645.1      ------GGKDRGAFAGDAGIASLERELNNMVRTNIDG---LDMTKFGITA----DKDGKL    337
                              . * : .*  :   :     :         ..     : .  **.      . *::

                                                                        FliD29
CBE01872.1          SFNADTFSKALIDDSDKVYKTLAGYSSNYDDKGMFEKLKDIVYEYSGSSTSKLPKKAGIE    445
WP_000146770.1      ELDADKLTAALKKDASGVGALIVGDGK---KTGITTTIGSNLTSWL-STT-------GII    404
WP_094313017.1      KIDDDKLKKVLNENTASVRELLVGDGK---ETGITTKIATEVKGYL-ADD-------GII    407
WP_057101173.1      EIDSDKFNEAMEANPDGLTSIFVGDDS------MVAQMDNLMDSYLDSST-------GII    379
WP_046449645.1      ELDSEKLEKMLTDNPTQLTALFNGNDG------LIKKMDKSLDKYLNSTN-------GVI    384
                    .:: :.:    :   :    :   : * .        :    :   :  :  :          *:

                        FliD30
CBE01872.1          KTASASENVYSKQIAEQERNISRLVEKMNDKEKRLYAKYSALESLLNQYSSQMNYFSQAQ    505
WP_000146770.1      KAATDGVSK---TLNKLTKDYNAASDRIDAQVARYKEQFTQLDVLMTSLNSTSSYLTQQF    461
WP_094313017.1      DSAQDSINA---TLKKLTKQYLSVSASIDDTVARYTAQFTQLDTMMSKLNNTSTYLSQQF    464
WP_057101173.1      TMREDNIED---QQSKIQDESDQLTETYNTNYERYLEEYTNTLIETYTMKVSMAAFM---    433
WP_046449645.1      KGRQESLDR---QETQLTDRSDKISTRYENSYNRYLKQFTQLQQVMSQMNNTMSMFGLV-    440
                         . .          :               :     *    :::          .       :

CBE01872.1          GN-----     507
WP_000146770.1      ENNSNSK     468
WP_094313017.1      TAMSNS-     470
WP_057101173.1      -------     433
WP_046449645.1      -------     440
```

Figure S3 Analysis of sequence homology of FliD proteins from different flagellated bacteria strains. NCBI strain annotations: CBE01872.1 *C. difficile*; WP_000146770.1 *E.coli*; WP_094313017.1 *S. sonnei*; WP_046449645.1 *H. alvei*; WP_057101173.1 *C. freundii*.
